# Characterizing Time-Series Roving Artisanal and Small-Scale Gold Mining Activities in Indonesia Using Sentinel-1 Data

**DOI:** 10.3390/ijerph19106266

**Published:** 2022-05-21

**Authors:** Satomi Kimijima, Masayuki Sakakibara, Masahiko Nagai

**Affiliations:** 1 Research Institute for Humanity and Nature, Kyoto 603-8047, Japan; sakaki@chikyu.ac.jp; 2 Graduate School of Science & Engineering, Ehime University, Matsuyama 790-8577, Japan; 3 Graduate School of Science and Technology for Innovation, Yamaguchi University, Ube 755-8611, Japan; nagaim@yamaguchi-u.ac.jp; 4 Center for Research and Application of Satellite Remote Sensing, Yamaguchi University, Ube 755-8611, Japan

**Keywords:** alluvial mining, artisanal and small-scale gold mining, Indonesia, landcover change, remote sensing, synthetic aperture radar

## Abstract

The rapid growth of roving mining camps has negatively influenced their surrounding environment. Although artisanal and small-scale gold mining (ASGM) is a major source of gold production, the mining activities and their activeness are not well revealed owing to their informal, illegal, and unregulated characteristics. This study characterizes the transformations of roving camp-type ASGM (R-C-ASGM) activities in Central of Katingan Regency, Central Kalimantan Province, Indonesia, from 2015 to 2021 using remotely sensed data, such as the time-series Sentinel-1 dataset. The results show that the growth of active R-C-ASGM sites was identified at the center of the Galangan mining region with expansions to the northwest part along the Kalanaman River, especially in 2021. Hence, these approaches identify the transformations of roving mining activities and their active or nonactive status even in tropical regions experiencing frequent heavy traffic rainstorms. They provide significant information on the socioenvironmental risks possibly caused at local and regional levels. Our results also inform the design of timely interventions suited to local conditions for strengthening environmental governance.

## 1. Introduction

The rapid growth of the rove-type mining sector has negatively influenced their surrounding environments. Therefore, detecting such occurrences, determining their development rate, and identifying their active or nonactive status should provide significant insights into identifying possible socioenvironmental problems caused at local and regional levels. This may also allow environmental governance to be promoted at various levels.

Artisanal and small-scale gold mining (ASGM) is a major source of gold production using rudimentary technology at individual or community levels despite being informal, illegal, and unregulated [1]. This sector has the largest employer in gold mining at the global level comprising 70% to 80% of informal small-scale workers [2]. Mercury is commonly used to increase the gold extraction process, resulting in highly toxic environmental and health risks due to mercury pollution throughout its emissions and release into water and the atmosphere, respectively [3,4,5]. Such mercury pollution has largely been observed in South America, Africa, and Asian regions. Indeed, environmental impacts, such as deforestation, geomorphic and hydrological changes [6,7,8,9,10,11], and health problems, such as mercury intoxication-oriented movement disorders and various injuries associated with the ASGM activities have been reported [12,13]. Despite its significant socioenvironmental impacts, more than 80 countries have continuously employed ASGM to alleviate poverty for their socioeconomic development [14,15].

Continuous growth has been observed in Indonesia. Active and nonactive ASGM practices have been placed in 93 regencies of 30 of the 34 provinces, estimating 250,000–300,000 miners [16] in more than 1200 hotspots in 2017 [17]. Furthermore, the country has been the fastest increase in polluted sites in the last 20 years on a global scale [2]. In Kalimantan island, one of the ASGM hotspots with alluvial operations, many illegal mining activities have been widespread even in conservation areas, impacting biodiversity and human health [17].

The ASGM sector can be classified into the following two types: “travel-type,” in which the miners commute from their local residences to the mining sites, and “camp-type,” in which the miners live and conduct mining activities on informal worksites [18] (hereafter referred to as C-ASGM). In the C-ASGM sector, both roving and non-roving practices are observed. The scale of the workforce in the ASGM sector has expanded with the increasing gold prices since 2000 [19]. The strong relationship between ASGM increases and the high price of gold has been confirmed in the literature [7,18,20].

Remote-sensing technologies have been widely used to characterize natural features and physical objects and monitor their spatial changes over time. Additionally, this technology provides a wide variety of continuous data with temporal, spatial, and spectral resolutions. Freely available satellite remote-sensing data, such as the Landsat series, have provided long-term Earth observation data since the 1970s and have been widely used for land cover detection and monitoring [21,22,23,24]. Despite the development in geoinformation technology, few studies have focused on the ASGM sector for quantitative assessments experiencing the harmful environmental and health risks caused by mercury pollution. Even [6,7,8,9,10,11,25,26,27] demonstrated time-series assessments in deforestation, mining area detection, and geomorphic and hydrological changes; however, they mainly examined the travel-type mining sites. To investigate the closed C-ASGM sites, Ref. [18] recently conducted a quantitative time-series analysis of the growth in C-ASGM sites using satellite remote-sensing imagery. Furthermore, Ref. [28] analyzed the transformation of C-ASGM activities by integrating nighttime light (NTL) intensities as a magnitude of mining activities. Although a time-series assessment of the closed C-ASGM sector with non-roving practices has been conducted by [18,28], a roving C-ASGM sector (hereafter R-C-ASGM) has not yet been discovered. The major challenges, such as acquiring an optical cloud-free time-series dataset [18,28,29], lead to further difficulty in understanding the R-C-ASGM sector, operated at a larger scale in tropical regions experiencing frequent heavy traffic rainstorms.

The use of the synthetic aperture radar (SAR), an active independent Earth observation system from solar illumination or day–night cycles [30], is an alternative suited tool for optical data [31]. Further, Ref. [32] reviewed the optical and SAR data for monitoring ASGM sites and ensured results between the datasets. Previous studies have revealed the potential of SAR data usage in mining-induced area detection using SAR sensitivities of radar systems to surface roughness and dielectric properties of materials [27,31,32]. Therefore, SAR data are a powerful tool to overcome weather-related limitations mainly found with optical sensors. This helps detect and monitor closed R-C-ASGM sectors to obtain a qualitative and comprehensive understanding.

This study primarily assesses the transformation of the R-C-ASGM activities from 2015 to 2021 in Katingan Regency, Central Kalimantan Province, Indonesia, where active alluvial-based R-C-ASGM activities have been conducted. This study’s results are expected to contribute to the understanding of R-C-ASGM development spread in remote rural areas, the prediction of the level of socioenvironmental pollution, and strengthening environmental governance at the regional level.

## 2. Materials and Methods

### 2.1. Overall Methodological Workflow

**Figure 1:**
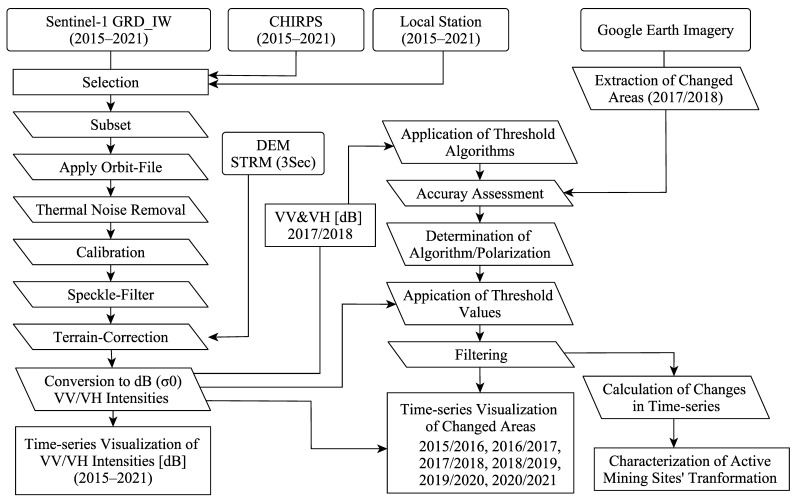
Overall methodology.

The methodological workflow used in this study is demonstrated in Figure 1. This workflow employed three main steps to achieve its primary objective of assessing the transformation of the R-C-ASGM activities. First, the S-1 backscattering coefficients (σ^0^) were calculated with vertical–vertical (VV) and vertical–horizontal (VH) polarizations. Second, selections of algorithm/polarization were performed to detect the most locally sensitive values. Third, the changes in the R-C-ASGM occurrences during 2015–2021 were calculated based on the S-1 temporal series. This evidence allowed us to understand the historical transformation of the R-C-ASGM activities at the study site. This study presents a discussion based on all the findings described above. The methods used in each step are explained in the following sections.

### 2.2. Study Area

Indonesia is a well-mineralized metallogenic region with significant gold mineralization, associated with quartz veins in andesite-hosted epithermal settings. One of the major ASGM hotspots in Central Kalimantan with gold-bearing alluvial soils has attracted large ASGM-targeted migrants from Java and South Kalimantan [33]. The Galangan mining region in Central Kalimantan is the geographical and historical center of the land-based mining area, which developed rapidly in the early 1990s [32]. The Hampalit town, especially, was a base for active mining activities for both indigenous miners and a gold company, namely PT Hampalit Mas Perdahana, which closed during the financial crash of 1997. Company-initiated mining activities extracted heavy minerals through an open-pit method, digging deep excavation pits. Thus, removing all the soil and vegetation landscape on the surface creates a barren wasteland [13,33]. However, indigenous ASGM communities have extracted gold along with river systems by floating pumps, resulting in disturbances of riverbanks and an increase in sediment volumes [33]. After the company’s closure, the lands were taken by migrated miners. They have continuously traveled to the greater areas, from Kalanaman, Pundu to Galangan, to explore newer locations with greater gold production by seasons [33].

**Figure 2:**
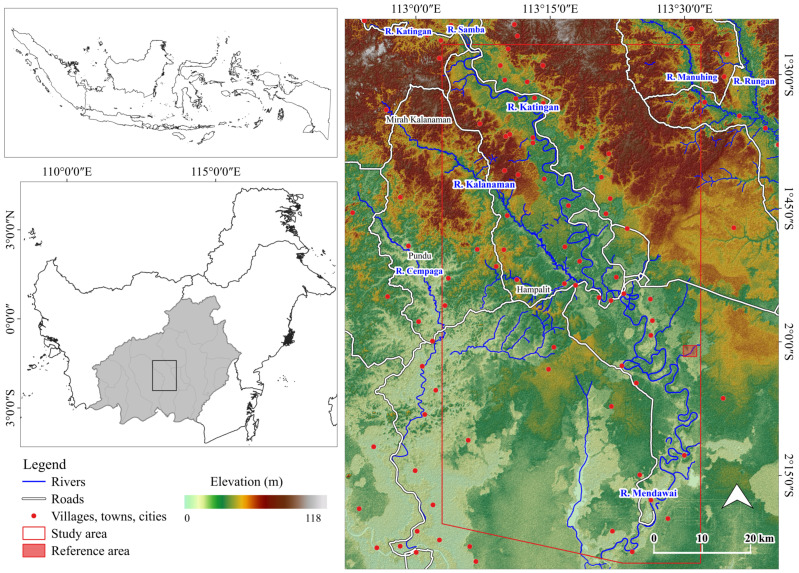
Study area.

This study targets Galangan mining (Central of Katingan Regency, the Central Kalimantan Province, Indonesia), utilizing the alluvial-based mining method (Figure 2). In this mining region, the Katingan River, one of the major river basins in Southeast Asia, flows north to south.

### 2.3. S-1 Imagery

A total of seven level-1 grand range detected (GRD) Sentinel-1 datasets, covering 2015–2021, downloaded from the European Space Agency (ESA), were utilized to extract and calculate time-series changes of the ASGM occurrences. Through the EU/ESA Copernicus program, the S-1 mission (S-1A and S-1B) provides an exceptional combination of high spatial (10 m) and temporal (6 days) resolution data by operating two polar-orbiting radar imaging systems working with the C band (~5.7 cm wavelength). The main operational mode is interferometric wide swath mode (IW) with VV and VH polarizations, and images are freely and routinely available [34].

To reduce atmospheric effects, which reduced the quality of images, Climate Hazards Group InfraRed Precipitation with Station (CHIRPS) data was referred to using Google Earth Engine to target months experiencing less rain with local weather station data. Thus, this study focused on July to August from 2015 to 2021.

All datasets were acquired from the descending track with relative orbit number 3 of each image’s backscatter intensity to better the image. The available S-1 dense time-series offers a unique opportunity to monitor ASGM activities, especially in tropical regions experiencing the magnitude of frequent rainstorms.

### 2.4. Image Preprocessing

The preprocessing workflow is based on ESA’s open-source software, ESA named sentinels application platform (version 8.0.0), and its functionalities. The following steps were implemented in the S-1 Toolbox: orbit correction, thermal noise removal, radiometric calibration, speckle filtering with 5 × 5 windows, and terrain correction using the 3-arcsec digital elevation model (DEM) from the shuttle radar topography mission (SRTM) [35]. Here, the radiometric calibration aims to convert the digital pixel value of the S-1 images into an image intensity value of σ^0^. The data were projected to the World Geodetic System 1984, Universal Transverse Mercator Zone 49 South. Terrain-corrected σ^0^ intensities of the VV and VH were used for further analysis.

### 2.5. Selection of Threshold and Detection of Changed Areas in Time-Series

After image preprocessing, optimized threshold values were identified based on the VV and VH polarizations acquired in 2017 and 2018. Sixteen automatic global thresholding algorithms and binary image classifications using one-dimensional feature space were applied to extract mining-induced areas. In this process, Fiji (version 2.1.0) software (https://imagej.net/software/fiji/, accessed on 1 March 2022), an open-source Java image processing package, was used to determine each algorithm’s threshold values. Huang’s fuzzy [36], Internodes [37], Isodata [38], IJ_Isodata, Li’s Minimum Cross-Entropy [39,40,41], Maximum Entropy [42], Mean [43], Minimum Error [44], Minimum [37], Moments [45], Otsu’s [46], Percentile [47], Renyi’s Entropy [42], Shanbhag’s [48], Triangle [49], and Yen’s [50] threshold algorithms were separately performed. This study also tested a supervised classification method, such as histogram intersection, applied by [31]. Subsequently, the results were validated using reference data to examine the best separability for the change detection. The reference data for the accuracy assessment were derived from high-resolution images obtained on 9 June 2017 and 23 September 2018, using Google Earth Pro.

Owing to heavy cloud coverage in the study area, the acquisition of the scenes was extremely limited only to the abovementioned data. However, these images identified mining activities along the Katingan River. According to human visual image interpretation, areas affected by mining activities were separately digitized, and the changed areas were identified by overlaying. Third, 100 points were randomly selected from the datasets to determine the best suitability by polarizations. Fourth, the determined best combination of algorithm and polarization was applied to all datasets post-classification of a majority filter with a moving window size of 5 × 5 pixels to remove isolated pixels. Furthermore, the detected areas observed in the river buffers were eliminated to remove the mudflats in the rivers, possibly caused by changes in the magnitude of precipitation between the acquired years. Consequently, the annual changes in the extent of illegal mining were calculated for the following six temporal series: 2015/2016, 2016/2017, 2017/2018, 2018/2019, 2019/2020, and 2020/2021.

In previous studies, mining areas in the Central of Katingan Regency were estimated to cover ~400 km^2^ in 2007 [32]. Hence, the long-term trends in R-C-ASGM sites could be observed from satellite imagery even with a 10-m ground resolution. We summarized the main specifications of the databases used in Table 1.

## 3. Results

### 3.1. Visualization of Time-Series Color Composites of VV and VH Polarizations

**Figure 3:**
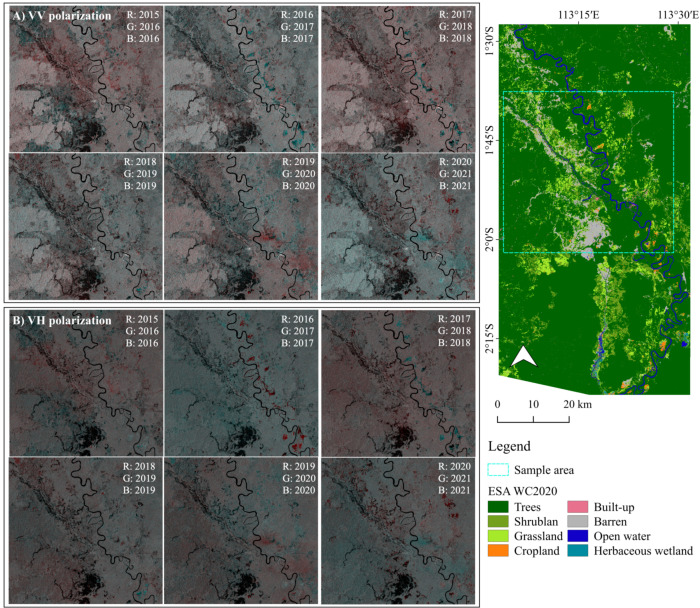
Time-series color composites by (**A**) VV and (**B**) VH polarization channels.

The processed VV and VH polarizations were displayed in RGB color composites in six temporal series: 2015/2016, 2016/2017, 2017/2018, 2018/2019, 2019/2020, and 2020/2021, as shown in Figure 3. In this visualization process, the older years were assigned red, and the newer years were assigned green and blue, which detects changes in land covers between two different periods. VV polarizations show slightly brighter intensities compared to that of VH’s. VH polarization can detect significant landcover changes along the Katingan and Kalanaman Rivers in 2016/2017 and 2020/2021.

### 3.2. Determination of Threshold

Both VV and VH polarizations acquired in 2017 and 2018 were primarily used to derive the best combination of algorithm and polarization. The changed areas identified from each result were validated using features extracted from high-resolution Google earth images (GEI), as mentioned in Section 2.5. The most sensitive algorithm and polarization channel indicate changes in illegal mining extents.

After processing the optimized thresholding, the locally sensitive methods were found only in the IJ_Isodata and Yen algorithms. Therefore, those were applied both to VV & VH polarization channels. Table 2 presents the identified thresholds. The threshold values identified by the IJ_Isodata showed lower intensities: −15.07 dB (2017_VV), −21.47 dB (2017_ VH), −14.84 dB (2018_VV), and −20.16 dB (2018_VH). The Yens were: −15.07 dB (2017_VV), −20.16 dB (2017_VH), 13.32 dB (2018_VV), and −20.16 dB (2018_VH). The results show no significant value differences in both VV and VH polarizations (IJ_Isodata algorithm). The same values were generated in 2017_VV and 2018_VH from the Yen algorithm; however, 2018_VV showed a larger difference between the two periods. As new mining areas are usually associated with land cover changes from vegetation to bare areas or water, the magnitude of intensity in such areas is expected to be lower intensities in VV and VH polarizations. Thus, the IJ_Isodata algorithm was more sensitive to finding mining activity-induced land landcover changes than the Yen algorithm in this study.

**Figure 4:**
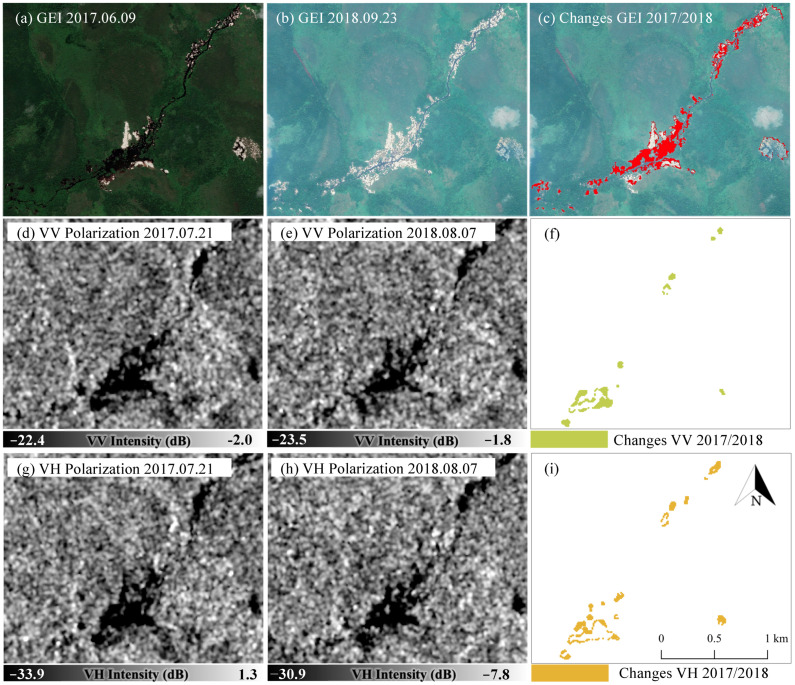
GEI 2017 (**a**), 2018 (**b**), detected changes from GEI 2017–2018 (**c**). VV polarization in 2017 (**d**), 2018 (**e**), detected changes from VV 2017–2018 after applying the threshold values (**f**). VH polarization in 2017 (**g**), 2018 (**h**), detected changes from VH 2017–2018 after applying the threshold values (**i**).

Figure 4 shows detected areas induced by R-C-ASGM activity during 2017/2018, based on human visual interpretation of GEI and thresholding results by VV and VH polarizations optimized by the IJ_Isodata algorithm. After different threshold values, similar intensities were found in both VV and VH polarization in the identified areas. For example, an average of −18.03 dB (standard deviation (STDEV) of 1.14 dB)) and −24.23 dB (STDEV of 1.51 dB) was observed for 2017 VV and VH, respectively. Furthermore, −18.11 dB (STDEV of 1.55 dB) and −23.89 dB (STDEV of 2.22 dB) were observed during 2018. By comparing the results, some areas in the middle part were not detected by VV and VH polarization; however, the visual comparison indicates that areas induced by R-C-ASGM activities can be detectable in both time-series features.

### 3.3. Detection of Newly Expanded R-C-ASGM Areas

Using the results in Section 3.1, the accuracy assessment was performed to judge their sensitivity. The results show 73.3% and 76.0% for the VV and VH polarizations, respectively. We recalculated the accuracy by omitting the points found at boundaries due to high spectral resolution sensitivity resulting from mixed pixels. As a result, we found 76.7% and 82.1% accuracies for the VV and VH polarizations, respectively. The best combination was found with the IJ_Isodata algorithm with VH polarization. The particular threshold values for each VH polarization were generated for the final classification, possibly leading to better detection of active R-C-ASGM activities (Table 3).

**Figure 5:**
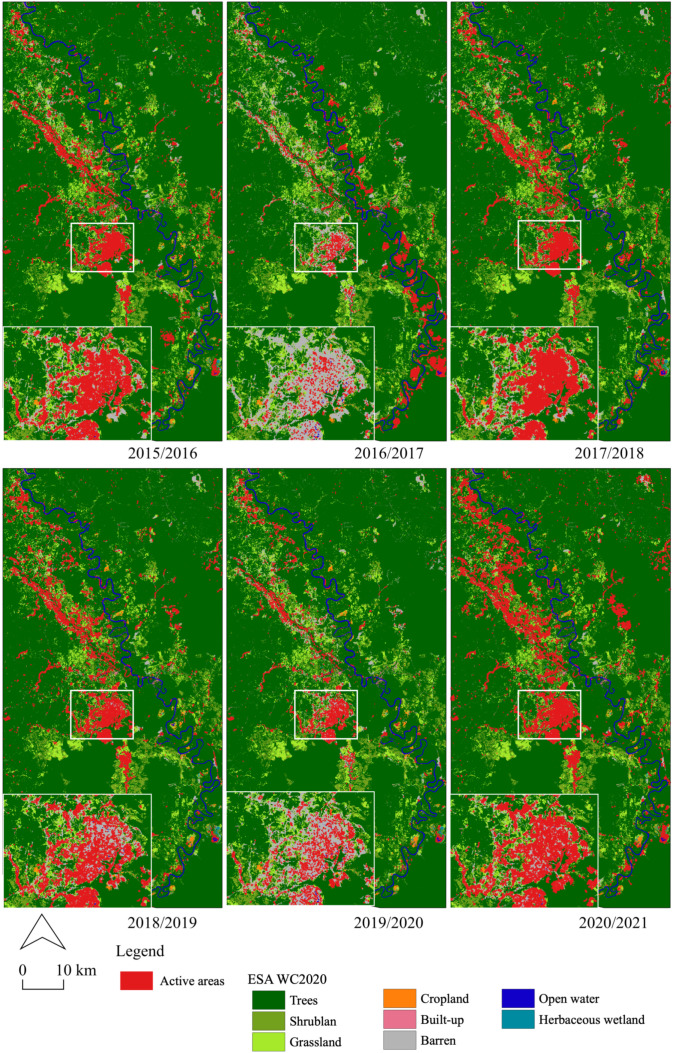
Occurrence of active mining sites detected by VH polarizations and their overlay on the ESA WC2020.

Figure 5 shows the occurrence of active mining sites for the six periods (2015/2016, 2016/2017, 2017/2018, 2018/2019, 2019/2020, and 2020/2021), overlaying on the European Space Agency (ESA) WorldCover 10 m 2020 (WC2020). The occurrence of R-C-ASGM-induced areas exhibited 25.0 km^2^ (2015/2016), 28.0 km^2^ (2016/2017), 32.1 km^2^ (2017/2018), 20.3 km^2^ (2018/2019), 7.4 km^2^ (2019/2020), and 47.9 km^2^ (2020/2021), respectively. The magnitude of the occurrences was found in 2015/2016–2017/2018; however, fewer occurrences were observed in 2019/2020. Simultaneously, the largest occurrence was again observed in 2020/2021 along the river. The detected areas were concentrated in the center of the Galangan region and along the Kalanaman River, where LC is classified as barren in ESA WC2020. The magnitudes of the occurrences were particularly observed in 2020/2021 in the northwestern parts of the study area along the Kalanaman River. The pattern of occurrences is observed mostly along with the river networks.

## 4. Discussion

### 4.1. Contributions

We studied the transformations of the R-C-ASGM activities from 2015 to 2021 using the S-1 time series. A quantitative time-series analysis of the R-C-ASGM sectors can help better understand the rate and pattern of development of such mining activities over time. Detecting such occurrences and their patterns in tropical regions experiencing the magnitude of frequent rainstorms can provide significant information or estimation on the potential rates and levels of socioenvironmental pollution and its human risk resulting from mercury use at R-C-ASGM sites. Understanding the characteristics of R-C-ASGM practices helps strengthen environmental governance at various levels.

As described, the establishment of new mining areas is usually associated with changes in land cover from vegetation to bare/water areas. We employed a change detection method based on generating the binary masks using a threshold defined by the image, optimized by the IJ_Isodata algorithm (Table 2). The analysis reveals that VH polarization was more sensitive than VV polarization, resulting in better separation of areas induced by mining activity (Table 3). While the classification accuracy was 76.0%, a higher accuracy (82.1%) was found with omission of random points at boundary. For 24.0% of errors, influence factors would not be caused by algorithm matter. Instead, the following factors can be considered for this misclassification; SAR specific errors such as foreshortening and layover in mountainous areas owing to the side looking of SAR; differences in data acquired time; weather conditions before the data acquired time; and spatial resolution of data. Previous studies using SAR datasets in the mining sectors only achieved 52.0% [51], 84.9% (producer accuracy), and 72.4% (user accuracy) [31]. Our study does not focus on generating a high-accuracy map of active mining, which can be a replacement for a field survey. Instead, we aim to provide information that leads to and supports the initial survey and social implementation at a local level. Without any field data, we cannot target any destinations for surveying, resulting in huge loss of time and cost. Thus, the generated possible active map helps plan field survey. Our study is not an alternative tool to a field survey; therefore, 76.0% (82.1% highest) accuracy is sufficient for this study.

This study demonstrated the transformation of active sites in the R-C-ASGM sector from 2015 to 2021 in the Galangan region, Central Kalimantan, Indonesia, where active alluvial-based R-C-ASGM activities have been historically conducted. This study detected the active mines and their various transformation forms using a quantitative analysis over time (Figure 5), as described in Section 3.2. Few studies have quantified R-C-ASGM practices with satellite imagery data. A recent study by [18,28] conducted a quantitative time-series analysis of the closed Non-R-C-ASGM sites, employing the vertical tunnel method (shaft) of mining, in Golontato, Indonesia, using optical satellite remote-sensing imagery. However, this work further quantified R-C-ASGM sites where activities are operated at a large scale in tropical regions experiencing frequent heavy rainstorms.

Few studies have utilized satellite data to reveal the volume of illegal mining activities. Especially, a recent study by [28] quantified that the extent of illegal mining sites and the magnitude of mining activities in the camps experienced 4.8- and 3.8-fold increases, respectively, from 2014 to 2020. Although the study areas, mining type, and indicator of transformations differ from this study, a similar trend in the occurrence of active illegal mining activities was found in their results. Further, Ref. [32] focused on a study area that is comparable to ours. Their study found an annual expansion of 8 km^2^ through the Landsat series in the Galangan region from 1999 to 2002 [32]. This study found a higher magnitude of the occurrence rate during 2015/2021. The possible reason for this may be associated with an increase in the global gold price since 2006. Similarly, the gold price in Indonesia has increased since 2007, with an especially steady increase since 2017, which approximately doubled at the end of 2020 [18]. The magnitude of occurrences was found especially in 2020/2021 when a rapid increase in the gold price was observed globally and nationally, while the lower rate of occurrence observed in 2019/2020 could be due to the globally spread influence of the coronavirus pandemic, which affects the workforce, mining activities, markets’ supply chain, and cash flow [52,53].

For the shifts in the occurrence patterns, the result showed that the magnitudes of active areas were found in the western Galangan region and along with the river networks in 2020/2021. Even migrated miners have continuously roved the greater areas by season [33]. Their main mining target sites can be shifted along the Kalanaman Rivers to explore greater gold production. The possible reason for this shift can be associated with the expansions of river extents. From the European Commission Joint Research Centre Yearly Water Classification History dataset [54], which contains yearly water classifications from 1988 to 2020, water extent along the Kalanaman River areas exhibited 1.83 (2015), 2.90 (2016), 2.91 (2017), 2.76 (2018), 2.59 (2019), and 3.40 km^2^ (2020), respectively. Thus, alternative R-C-ASGM sites were expected to be further developed that were associated with the expansion of water extents along the Kalanaman River after 2020. Moreover, Ref. [32] previously revealed shifts in mining direction with PALSAR (June-September 2006). The authors found the various shifts in the active area of the western Galangan region. However, a time-series analysis of the study further identifies the detailed characteristics such as volumes, mine status, and trends of active mins’ shifting directions in the hidden R-C-ASGM sectors, representing a more comprehensive understanding of R-C-ASGM sectors across the region.

The R-C-ASGM sector can operate successfully due to its high productivity of gold. However, it is estimated that approximately 270 tons of mercury are annually released only from Central Kalimantan to the Sea of Java as of 2007 [32]. Furthermore, severe mercury contamination (sediment, local fish, and hair samples) and typical symptoms resulting from mercury intoxications (ataxia, tremor, and dysdiadochokinesia) among workers with high ASGM activities have been observed in the Galangan region [13]. Despite its status as an informal sector, the increase in global gold prices accelerated the massive entry of immigrants into the mining sector, resulting in its massive growth. The growth in those sectors further accelerate to cause mercury-related environmental pollution and health problems at the stages of mining and amalgamation. Therefore, detecting such rapidly developing hidden R-C-ASGM sectors can provide significant insights into the potential rates and the levels of socioenvironmental pollution. This would also strengthen the environmental governance with the participation of different stakeholders at various levels.

### 4.2. Limitations

This study has certain limitations associated with the characteristics of SAR data. Although SAR data helps in the active independent observation of weather, it causes foreshortening and layover in mountainous areas owing to the side looking of SAR, leading to misclassification. Further, precipitation before the acquisition can decrease the backscatter intensity in polarizations, overestimating illegal mining extents. Moreover, some smaller and complex areas are undetectable due to the used datasets (10 × 10 km grid cell). Finally, because of the S-1 series’ operation period, the methodologies applied in this study are limited only to the period after 2014.

Although the proposed method cannot detect the existing mining areas before 2015, it identifies the occurrences of R-C-ASGM-related active sites and their changing patterns.

## 5. Conclusions

This study assessed the transformation of the R-C-ASGM sector in Katingan Regency, Central Kalimantan Province, Indonesia, using S-1 time-series data. The results presented herein show the massive occurrence of the active R-C-ASGM sites. In particular, a magnitude of occurrence was found in the center of the Galangan region and along the Kalanaman River in 2021. Therefore, it can be concluded that the active mining sector undertaken by the R-C-ASGM method can be detected from a set of time-series datasets. These results extend our understanding of the transformations of the mining site and the status of their activeness in the hidden R-C-ASGM sectors. Subsequently, it also provides significant insight into the potential for further socioenvironmental problems at the regional level. These findings are expected to assist in developing rapid and appropriate interventions for strengthening environmental governance by involving various stakeholders.

## Figures and Tables

**Table 1:** Main specification of satellite imagery used in the study.

Satellite	Type	Acquisition Date	Spatial Resolution	Image Number	Polarization	Wavelength
Sentinel-1	C-SAR	20 July 2015 7 August 2016 21 July 2017. 4 July 2018. 11 July 2019 10 August 2020 24 July 2021	10 m	3	Descending (VV, VH)	C band

**Table 2:** Threshold values by algorithm and polarizations.

	2017		2018	
Algorithm	VV	VH	VV	VH
IJ_Isodata	−15.07 dB	−21.47 dB	−14.84 dB	−20.16 dB
Yen	−15.07 dB	−20.16 dB	13.32 dB	−20.16 dB

**Table 3:** Threshold values for time-series VH polarizations.

Threshold (IJ_Isodata Algorithm)	2015	2016	2017	2018	2019	2020	2021
Intensities (dB)	−20.88	−19.95	−21.47	−20.16	−20.36	−20.76	−19.8

## Data Availability

Not applicable.
